# Motif kernel generated by genetic programming improves remote homology and fold detection

**DOI:** 10.1186/1471-2105-8-23

**Published:** 2007-01-25

**Authors:** Tony Håndstad, Arne JH Hestnes, Pål Sætrom

**Affiliations:** 1 Department of Computer and Information Science, Norwegian University of Science and Technology, NO-7052, Trondheim, Norway; 2 Interagon AS, Laboratoriesenteret, NO-7006 Trondheim, Norway

## Abstract

**Background:**

Protein remote homology detection is a central problem in computational biology. Most recent methods train support vector machines to discriminate between related and unrelated sequences and these studies have introduced several types of kernels. One successful approach is to base a kernel on shared occurrences of discrete sequence motifs. Still, many protein sequences fail to be classified correctly for a lack of a suitable set of motifs for these sequences.

**Results:**

We introduce the GPkernel, which is a motif kernel based on discrete sequence motifs where the motifs are evolved using genetic programming. All proteins can be grouped according to evolutionary relations and structure, and the method uses this inherent structure to create groups of motifs that discriminate between different families of evolutionary origin. When tested on two SCOP benchmarks, the superfamily and fold recognition problems, the GPkernel gives significantly better results compared to related methods of remote homology detection.

**Conclusion:**

The GPkernel gives particularly good results on the more difficult fold recognition problem compared to the other methods. This is mainly because the method creates motif sets that describe similarities among subgroups of both the related and unrelated proteins. This rich set of motifs give a better description of the similarities and differences between different folds than do previous motif-based methods.

## Background

A huge gap exists between the number of protein sequences and the number of proteins with a known structure and function. The exponential growth in sequence information means that better methods to automatically annotate new sequences are needed. Current methods include *ab initio *structure prediction, sequence-structure comparisons, and sequence comparisons [1,2]. *Ab initio *methods try to predict the native protein structure from the amino acid sequence. The protein can then be annotated by comparing the predicted structure to those of proteins with known structure and function. Sequence-structure comparisons, or threading methods, try to fit the protein sequence to known structures. Compared with *ab initio *predictions, threading limits the candidate solutions to those structures already known. Sequence comparisons are based on the assumption that similar sequences share a common ancestor – that is, they are remote homologues – suggesting structural and functional similarities. Several good solutions exist when the level of sequence similarity is high, but when the sequences are highly divergent it is still difficult to distinguish remotely homologue sequences from sequences that are similar by chance.

Early solutions to the problem of finding remote homologues, such as the Smith-Waterman algorithm [3] and heuristic alternatives like BLAST [4] and FASTA [5], looked for sequence similarity between pairs of proteins. Later solutions used aggregated statistics of related proteins to generate more complex models that a protein with unknown function could be compared to. These methods, including profiles [4,6] and hidden Markov models (HMMs) [7-9] used only related sequences for model generation.

The most successful recent methods have been discriminative. Classifiers are trained on both related and unrelated proteins to recognize what distinguishes the related proteins from the unrelated ones. Kernel methods such as the support vector machine [10] have proven to give particularly good results, and several groups have introduced different types of kernel functions [11-19]. Most of these kernel functions are typically either based on profiles and sequence alignments, or based on the occurrences of discrete motifs.

### Kernels based on profiles and sequence alignments

The Fisher kernel [11] was the first method that used support vector machines. This method trains profile HMMs on related proteins and produces feature vectors from sequences by aligning them to the HMMs. Another alignment-based kernel is SVM-Pairwise [12], which represents each sequence as a vector of pairwise similarities to all sequences of the training set. The SVM-I-sites method [13] compares sequence profiles to the I-sites library of local structural motifs for feature extraction and this method has also been improved to take into account the order and relationships of the I-site motifs [14].

A relatively simple but efficient kernel is the Mismatch kernel [15] in which the feature space consists of all short subsequences of length *k*, called *k*-mers. A *k*-mer is said to be present in a sequence if the sequence contains a substring that has at most *n *mismatches to the *k*-mer. In the profile kernel of Kuang et al. [16], the mismatch kernel is combined with profiles; a *k*-mer is said to be present in a sequence if the sequence contains a substring that when aligned to the profile gives a score above a given threshold. Later methods, such as the LA-kernel [17] and SVM-SW [18] are also alignment-based, but instead of representing the sequences as a vector of features they calculate the kernels directly by an explicit protein similarity measure. The LA-kernel uses all optimal gapped local alignment scores for all possible subsequences of two sequences, while SVM-SW uses the optimal local alignment that maximizes a direct profile-profile score.

### Kernels based on discrete sequence motif content

Motif kernels are based on the idea of using motif content to measure sequence similarity. Protein sequence motifs describe some common sequence pattern that is conserved over greater evolutionary distance than the rest of the sequences. Focusing on sequence motifs therefore means focusing on the most conserved parts of a sequence, where remote homologues are most likely to share similarities.

Although there are many databases of sequence motifs available [20-23], these databases were created in a supervised way to have motifs that characterize different known protein families, domains, or functional sites. Consequently, a motif kernel based on these databases will be biased towards correctly classifying known functions or families. This also makes such motif kernels inappropriate in benchmark studies. The eMOTIF kernel of Ben-Hur and Brutlag [19] avoids these problems by using motifs extracted with the unsupervised eMOTIF method [24] from the eBLOCKS database [25]. The eMOTIF kernel has good performance when classifying sequences in classes for which several motifs are available, but the performance decreases when related sequences share few or no motifs [16].

An alternative to using motifs from an existing database is to generate the motifs from the available data. We introduce a motif kernel where genetic programming is used to find discriminative sequence patterns matching the positive training set sequences while not matching the negative training set sequences. The motifs are made from a simple regular expression-like grammar and the resulting matches against the data set is used to build feature vectors for a support vector machine.

We benchmark our GPkernel on updated versions of two commonly used benchmarks [18] based on the SCOP database [26] and compare its performance with the eMOTIF, Mismatch, SVM-Pairwise, and LA kernels as well as the PSI-BLAST method. We find that our method achieves performance similar to the LA-kernel method and gives significantly better results than all of the other methods. We also find, when comparing the GPkernel to related motif methods, that motifs trained on the different classes of negative sequences are vital for the method's predictive power.

## Results and discussion

### Genetic programming for protein motif discovery

There are several methods that use genetic programming (GP) [27] to evolve Prosite motifs [20] from multiple unaligned [28-30] or aligned [31] sequences. Genetic programming has also been used to create stochastic regular expression motifs [32]. We use GP to evolve the discrete sequence motifs that serve as a basis for our methods.

Our GP algorithm is trained on a positive and negative training set and the fitness of a candidate solution is a function of its matches in the two sets. More specifically, the fitness is

f=12(TPTP+FN+TNTN+FP)=12(Se+Sp),     (1)
 MathType@MTEF@5@5@+=feaafiart1ev1aaatCvAUfKttLearuWrP9MDH5MBPbIqV92AaeXatLxBI9gBaebbnrfifHhDYfgasaacH8akY=wiFfYdH8Gipec8Eeeu0xXdbba9frFj0=OqFfea0dXdd9vqai=hGuQ8kuc9pgc9s8qqaq=dirpe0xb9q8qiLsFr0=vr0=vr0dc8meaabaqaciaacaGaaeqabaqabeGadaaakeaacqWGMbGzcqGH9aqpdaWcaaqaaiabigdaXaqaaiabikdaYaaadaqadaqaamaalaaabaGaeeivaqLaeeiuaafabaGaeeivaqLaeeiuaaLaey4kaSIaeeOrayKaeeOta4eaaiabgUcaRmaalaaabaGaeeivaqLaeeOta4eabaGaeeivaqLaeeOta4Kaey4kaSIaeeOrayKaeeiuaafaaaGaayjkaiaawMcaaiabg2da9maalaaabaGaeGymaedabaGaeGOmaidaaiabcIcaOiabdofatjabdwgaLjabgUcaRiabdofatjabdchaWjabcMcaPiabcYcaSiaaxMaacaWLjaWaaeWaaeaacqaIXaqmaiaawIcacaGLPaaaaaa@524B@

where TP, FP, TN, and FN are the number of true and false positives and true and false negatives, and *Se *and *Sp *are the rates of correctly classified positives and correctly classified negatives. These rates are also known as the sensitivity and specificity. The fitness evaluation is accelerated by special purpose search hardware [33], which reduces the training time.

The hardware supports several regular expression-like operators, but we use only a small subset of these. Our solution language is formally defined for the DNA alphabet elsewhere [34], but is here modified to handle the protein alphabet of amino acids. The amino acid symbols are the language's basic elements. The language then uses several operators to build patterns from the basic amino acid letters.

Concatenation is the simplest operator and it gives patterns that match sequential occurrences of amino acids; for example, the pattern *GLAA *matches the sequence GLAA. The logical disjunction operator allows alternative amino acids at specific positions, such that the pattern *GL (A|C) A *matches sequences GLAA and GLCA. The wildcard operator is a special variant of the logical disjunction operator as it matches any amino acid. Thus, the pattern *GL.A *matches GLAA, GLCA, GLDA, and so forth.

The final operator, called the "p-of-n" operator, can be used to specify the minimum number of amino acids that must match within its sub-pattern. To illustrate, the pattern {*GLAA *: *p *≥ 3} will match GLAA, GCAA, and ELAA, but not match GLCC, and GCAQ. Note that the *p *defines the minimum number of amino acids that must match. The "p-of-n" operator is also called the Hamming distance operator, as it matches sequences that have a Hamming distance below *n *– *p*, where *n *is the sub-pattern's number of amino acids.

By using the Hamming distance operator, we can specify patterns that have a certain number of mismatches. Furthermore, by combining the Hamming distance and the logical disjunction operators, we can boost the importance of amino acid residues at specific positions in the pattern. To illustrate, the pattern {*G*(*L*|*L*)*AA *: *p *≥ 4} gives a double weight to Leucine at the second position. This pattern will only match sequences that contain Leucine and at least two of the three other amino acids, such as ALAA, GLCA, and GLAE. We can use this position specific weighting of amino acids to define patterns that approximate position weight matrices.

### The GPkernel uses diverse motifs

The problem of protein remote homology detection has an inherent structure as all proteins can be grouped according to evolutionary relations and structure. This is the basis for the SCOP hierarchy [26]. Our method uses this inherent structure to create a kernel based on a rich set of motifs that tries to capture information about all related sequences in a particular dataset.

To compute the kernel, we use the GP algorithm to produce motifs from the SCOP training set. The kernel is therefore referred to as the GPkernel. For each superfamily classifier, we make both positive and negative motifs; that is, motifs trained to match the classifier's superfamily as well as motifs trained to match the other superfamilies. Correspondingly, we do the same for the fold benchmark and train motifs for all folds. This is done based on the hypothesis that motifs trained to recognize the different aspects of the negative data will increase the discriminative power of the GPkernel.

**Figure 1:**
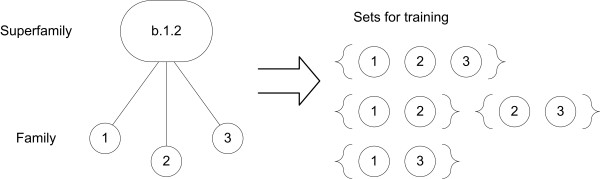
**Splitting of training sets for GP algorithm**. The figure shows three families from an example superfamily that constitute the positive training set for GP. The positive test set and the negative training and test set are not shown in this figure. In addition to train motifs to cover the sequences of all three families, we also train GP on all the possible subsets of this superfamily that exclude one family of the positive training set. This is indicated by the four sets in the right part of the figure. Ten motifs are made for each subset.

The basic positive training sets for GP include all members of a superfamily or fold, except for the sequences forming the positive test set. This means that all the motifs will have to cover a large taxonomic distance. To narrow the structural range each motif has to cover, we also split the positive training set into subsets, as shown and explained in Figure 1. For the superfamily benchmark, the subsets exclude one family from the superfamily sequences. For each such subset, we make ten motifs. We also make ten motifs trained on all the sequences of the superfamily. As a consequence, the number of motifs produced for each superfamily will be ten times the number of families in the superfamily. In total, this produces 3350 motifs for each classifier in the superfamily benchmark.

For the fold benchmark, it is not practical to generate motifs for all subsets excluding one superfamily because of the very high number of superfamilies in each fold. As we still want several motifs representing different subsets within a fold, we adopt a slightly different solution. The sequences of a fold are grouped into superfamilies and ten sets are made for each fold such that each set exclude one tenth of the sequences. This gives 3300 motifs for each classifier in the fold benchmark.

For both benchmarks, we run GP with a population of 100 candidate solutions for 50 generations. The resulting motifs are matched against all the sequences to produce a matrix of binary feature vectors. This matrix contains a 1 at position (*i*, *j*) if sequence *i *contains motif *j *and a 0 otherwise. The Gram matrix is then produced by taking the dot product between the vectors of the sequences; see Methods for additional details.

As described above, training the GPkernel basically amounts to 1) training motifs on each superfamily and fold for the respective problems, 2) joining the motifs into a common kernel, and 3) training an SVM to recognize each of the superfamilies or folds. Although this is how one would train final versions of classifiers for predicting superfamily or fold membership, training one set of motifs from the complete database is not appropriate for a benchmark study that tries to assess the method's predictive power. When estimating the predictive performance of any machine learning method, it is essential that one evaluates the trained models on test sets that are independent of the training set; that is, none of the sequences in the test set should be part of the training set. Otherwise, the performance estimates will be biased. To ensure that our performance estimates are unbiased, we therefore create a separate set of motifs for each of the superfamily and fold test sets. Consequently, we create 102 * 3350 = 341, 700 and 86 * 3300 = 283, 800 superfamily and fold motifs for the SCOP 1.67 benchmarks, which means we will have evaluated about 3 * 10^9 ^GP patterns. Each GP run takes under 2 seconds on our Pentium 4 2.8 GHz system accelerated by one search chip. This gives a total runtime of about 14 days for both SCOP 1.67 benchmarks, but as we run several parallel processes on different subsets of the benchmarks, we can reduce the evaluation time to get complete results within one day. As a simple comparison, nrgrep [35] uses about 1 minute to evaluate 5000 expressions with varying numbers of amino acids and mismatches.

### Boosted classifiers and an extended eMOTIF kernel

**Figure 2:**
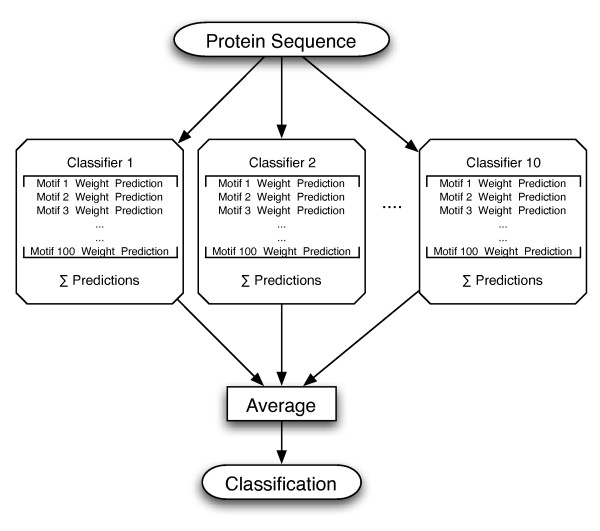
**Boosted classifiers**. The figure shows the setup of the boosted classifiers. For each test set, we create 10 boosted classifiers whose predictions are averaged to give a final classification. Each classifier is made from 100 boosted GP motifs.

The GPkernel uses motifs made from genetic programming as a basis for a support vector machine kernel. We also propose another method in which we use the GPboost program [34] to build boosted classifiers [36]. Each such classifier is based on 100 weighted sub-motifs where each sub-motif is made by running genetic programming on the SCOP training sets with a population of 100 candidate motifs for 100 generations. A boosted classifier's prediction is a sum of the predictions from the weighted sub-motifs. In addition, 10 boosted classifiers are made and combined so that the final prediction for a new sequence will be the average of 10 boosted classifiers. The setup is explained in Figure 2.

The eMOTIF kernel has shown good performance on protein families that share many eMOTIFs, but the performance decreases for families that are not covered well by the eMOTIFs. We propose to extend the eMOTIF kernel with an additional small set of GP motifs in hope that this will give a better performance when classifying the sequences that share fewer eMOTIFs. The extended eMOTIF kernel, called GPextended, is made from an eMOTIF kernel with additional GP motifs trained to target the positive training set. The motifs are made in exactly the same way as for the GPkernel, but only the positive motifs – the motifs trained on the subsets of a given classifier's training set – are added to an eMOTIF kernel to create the GPextended kernel.

### The GPkernel performs significantly better than the other motif-based methods

**Figure 3:**
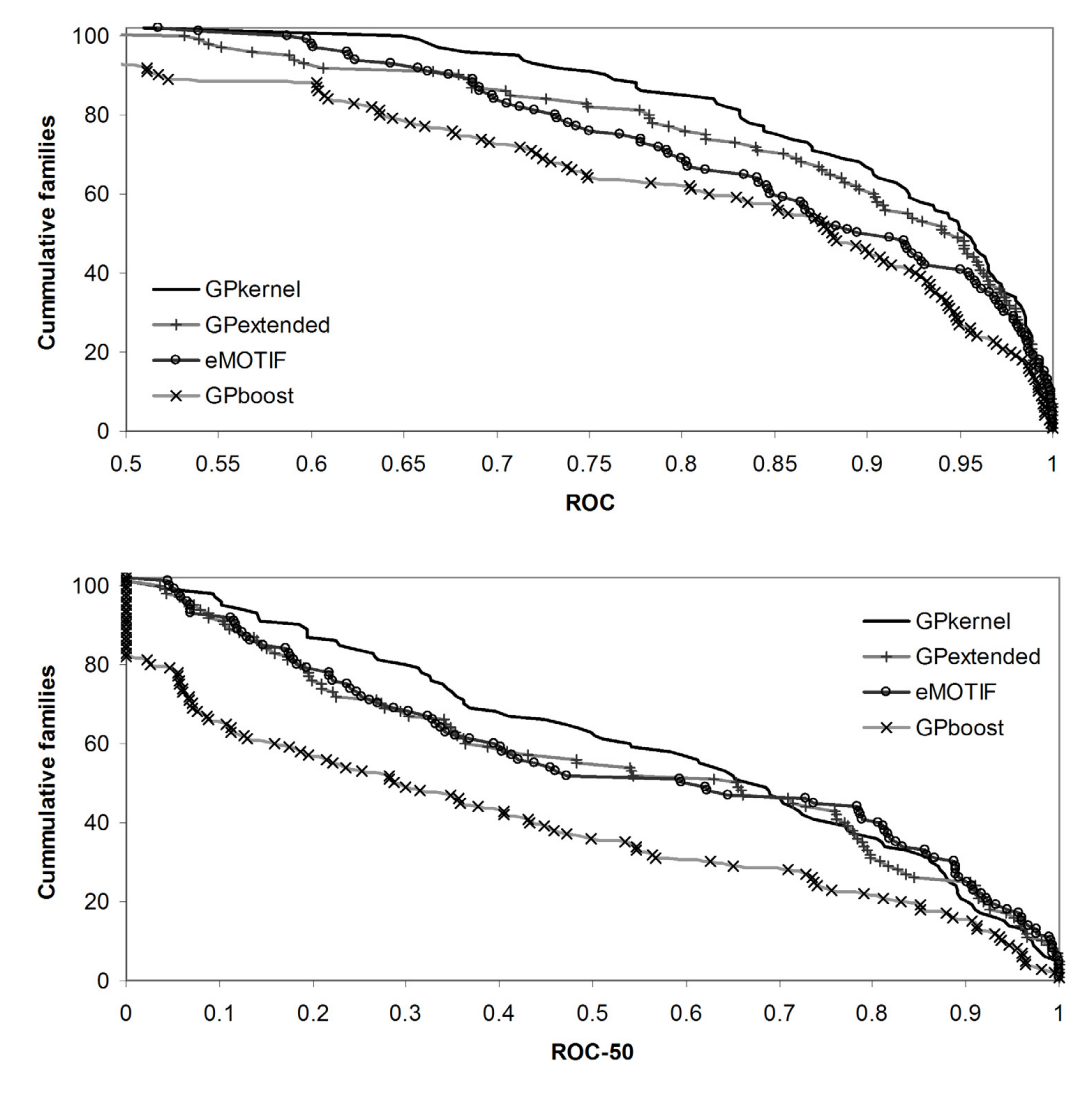
**GPkernel has highest overall performance of motif methods on superfamily benchmark**. The graphs show the cumulative number of families with a ROC (top) and ROC-50 (bottom) score greater than a given value for the GPkernel, GPboost, GPextended, and eMOTIF methods.

Figure 3 shows the performance of the GPkernel, GPboost classifier, the eMOTIF kernel, and the GPextended kernel on the superfamily benchmark. The GPkernel has the best overall performance in terms of both the cumulative ROC and ROC-50 scores, and the differences to the eMOTIF kernel are significant for the cumulative ROC-score (*p *= 4 * 10^-4 ^and *p *= 0.7 on ROC and ROC-50 results with signed rank tests corrected for multiple testing). The results also indicate that extending the eMOTIF kernel with GP motifs improves the performance of the eMOTIF kernel. Even though the performance differences between the GPextended and eMOTIF kernel are not significant (*p *= 0.3 and *p *= 0.9 on ROC and ROC-50), the GP motifs boost the performance of the eMOTIF kernel such that the GPextended kernel's ROC-scores are not significantly worse than those of the GPkernel (*p *= 0.06).

**Figure 4:**
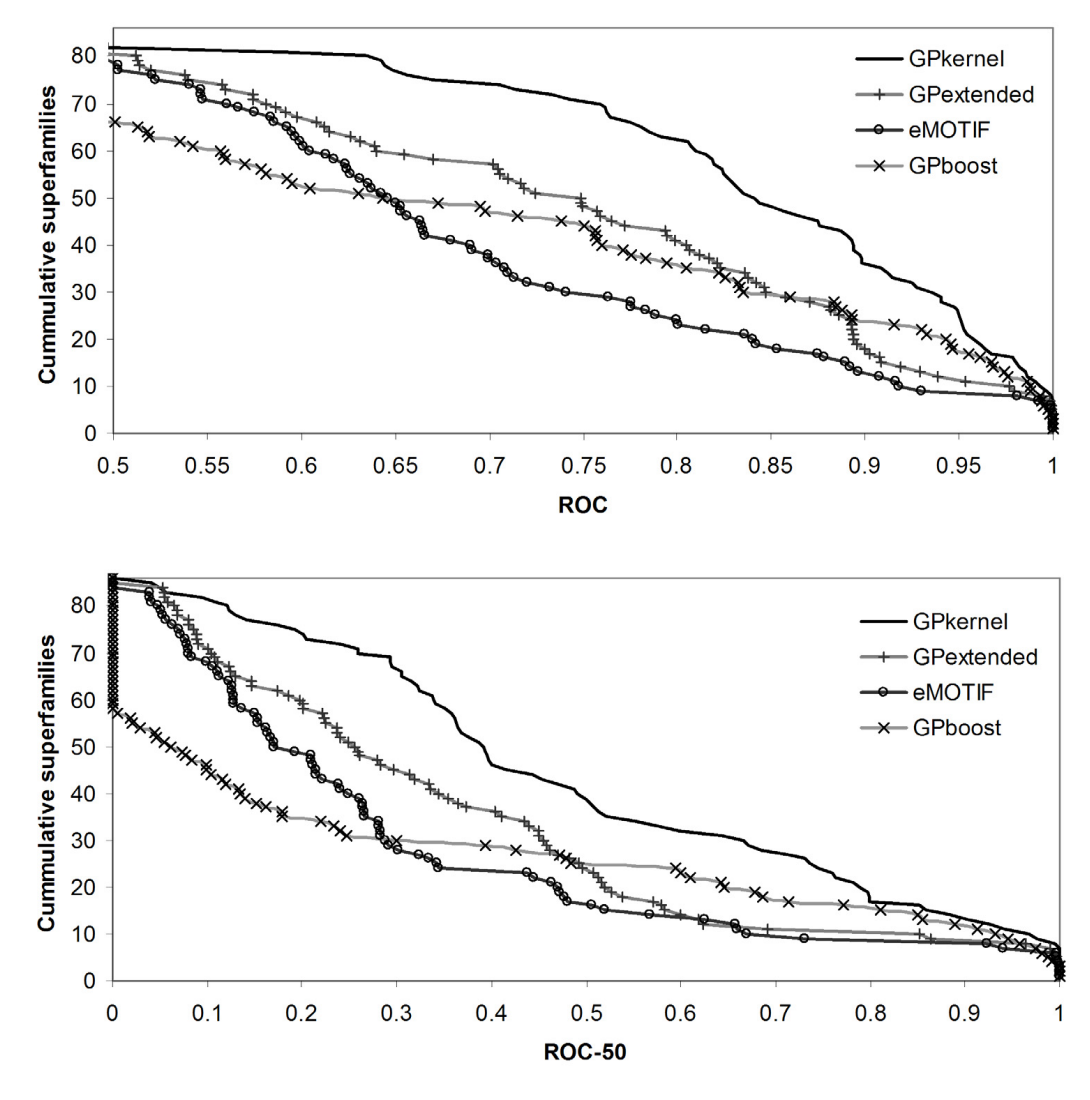
**GPkernel outperforms other motif methods on SCOP fold benchmark**. The graphs plot the cumulative number of superfamilies with a ROC (top) and ROC-50 (bottom) score greater than a given value for the GPkernel, GPboost, GPextended and eMOTIF methods.

As Figure 4 shows, the gain of adding additional motifs to the eMOTIF kernel is more evident on the fold benchmark. Because most of the eMOTIFs are relatively specific, the sequences that belong to a fold will on average share few eMOTIFs, giving a very sparse kernel. This might explain the huge performance drop for the eMOTIF method compared with its performance on the superfamily benchmark. If the eMOTIF method lacks a suitable set of eMOTIFs for fold detection, the additional motifs made for the GPextended kernel can compensate for this. Both the GPextended kernel's ROC and ROC-50 scores are significantly better than the eMOTIF scores (*p *= 2 * 10^-4 ^and *p *= 2 * 10^-4^).

The GPkernel has a very good performance on fold detection compared to the other motif methods (all *p*-values ≤ 10^-8^). The key to the GPkernel's increased performance are the motifs trained on the different negative folds. When we tested a kernel that consisted of an equal number of positive motifs only, the average ROC-50 score fell by 30% (data not shown). Similarly, GPboost and the GPextended kernel only use motifs trained to recognize the positive training set and are less accurate than the GPkernel is. As the above experiments have shown, the negative motifs are more useful on the fold than on the superfamily recognition problem. Because the positive sequences are more similar on the superfamily than on the fold benchmark, methods that only focus on recognizing the positive sequences can more easily find motifs that characterize the positive sequences than they can on the fold benchmark. On the fold benchmark, the motifs that characterize the positive sequences do not confidently predict a protein's correct fold, but an absence of motifs common to some of the negative folds may complement the occurrence of positive motifs. This complementarity probably explains the GPkernel's higher relative performance on the fold than on the superfamily benchmark compared to the other motif methods. To further put the GPkernel's performance in perspective, we benchmarked the GPkernel on Liao and Noble's SCOP 1.53 superfamily benchmark set [12,37]. The only difference from their original benchmark to our setup is that we randomly assign individual families instead of individual sequences to the test set. Table 1 summarizes the GPkernel's average ROC-score on the 54 test sets and compares this average to the averages of the other motif-based methods that have been benchmarked on the set. We have also included a recent method that uses latent semantic analysis (LSA) on three different motif kernels [38]. Dong and colleagues showed that using LSA on the best motif kernel, which consisted of *χ*^2 ^selected patterns extracted by the TEIRESIAS algorithm [39], gives a performance comparable with the performance of the LA-kernel [17]. Both the basic and LSA-optimized kernels (SVM-Pattern and SVM-Pattern-LSA; Table 1) have lower average ROC-scores on the SCOP 1.53 superfamily benchmark than the GPkernel has.

**Table 1:** Average ROC scores for motif-based kernels on SCOP 1.53 superfamily and fold benchmarks

Method	SF	Fold	Source
GPkernel	0.899	0.825	
Mismatch	0.872	-	[17,18]
SVM-Pattern [38]	0.835	-	[38]
SVM-Pattern-LSA [38]	0.879	-	[38]

LA-eig (*β *= 0.2)	0 923	0 847	[18]
LA-eig (*β *= 0.5)	0.925	0.771	[18]
SW-PSSM(3.0,0.750,1.50)	0.982	0.933	[18]
BV-PSSM(4)	0.963	0.941	[18]

SF and Fold are the average ROC-scores on the SCOP 1.53 superfamily and fold benchmark sets [12,18]; dash (-) represents an unreported value; Source is the sources of results other than ours. For reference, the table's lower part shows the best average ROC-scores reported by Rangwala and Karypis [18]. These methods do not use discrete sequence motifs.

Apart from the LSA, Dong and colleagues' approach differs from ours in that the TEIRESIAS algorithm is an unsupervised approach that finds all patterns occurring more than a specified number of times in the input sequences. Although this approach should give a similar good coverage of the complete training set as the GPkernel, the feature selection biases the initial set towards patterns that discriminate between the positive and negative training sets. This procedure also likely removed the patterns that discriminate between the different subgroups in the negative set. Our results show that such patterns are crucial for the GPkernel's performance.

The SCOP 1.53 benchmark set differs from our SCOP 1.67 set in two ways. First, Liao and Noble used a slightly more stringent criterion to filter out similar sequences. Second, and more importantly, the set includes all sequences that pass the similarity filter, whereas our set only includes sequences from superfamilies that have at least one family with ten or more sequences and one or more additional families that together have at least ten sequences. The result is that for our benchmark set, the GPkernel consists of motifs from all superfamilies, whereas for the SCOP 1.53 set, some superfamilies will not have any motifs. Thus, if the negative motifs are important for the GPkernel's performance, the GPkernel should have a lower performance on the SCOP 1.53 set than on the 1.67 set. Nevertheless, the average ROC scores on the two sets are almost identical (0.899 and 0.902 on SCOP 1.53 and 1.67). Similarly, when we benchmarked the GPkernel on Rangwala and Karypis' SCOP 1.53 fold benchmark [18], the GPkernel's average ROC score was 0.825; the average ROC score on the SCOP 1.67 benchmark was 0.844. Although results on the SCOP 1.53 and 1.67 cannot be compared directly, these results suggest that the negative motifs may not be that important for the GPkernel's performance, which is contrary to what the initial results suggest. The average ROC-50 scores on the superfamily and fold benchmarks show, however, that the negative motifs are important. Even though the GPkernel's overall performance is comparable on the two benchmark sets, the GPkernel has a large drop in average ROC-50 score on the SCOP 1.53 set (from 0.591 and 0.514 to 0.265 and 0.111 on superfamily and fold). This large drop is likely caused by the GPkernel missing negative motifs from some of the superfamilies and folds, which then led to these superfamilies and folds being overrepresented as false positives among the high-scoring sequences.

### The GPkernel has better overall performance than most existing methods

**Figure 5:**
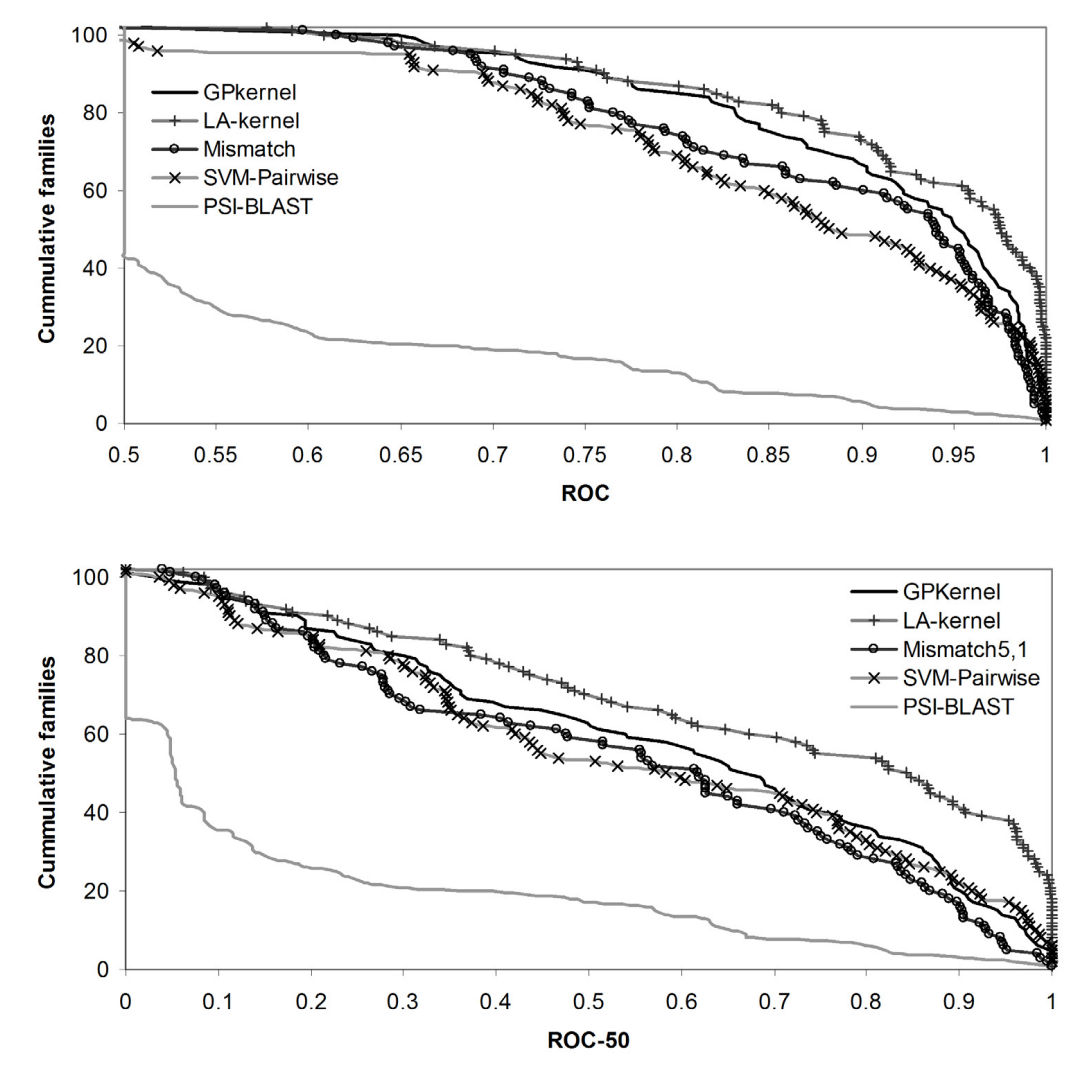
**GPkernel compares favorably to some common methods for remote homology detection on superfamily benchmark**. The figure plots the cumulative number of families with a ROC (top) and ROC-50 (bottom) score greater than a given value for the GPkernel, LA-kernel, Mismatch, SVM-Pairwise and PSI-BLAST methods.

To further assess the GPkernel's performance, we evaluated the performance of four other popular methods for remote homology detection: PSI-BLAST and the LA-kernel, Mismatch, and SVM-Pairwise kernels. Figure 5 summarizes the performances of the five methods on the superfamily benchmark. The GPkernel is significantly better than the other methods, except the LA-kernel, in terms of ROC scores (*p*-values of 0.001, 0.0004, and < 10^-10 ^for Mismatch, SVM-Pairwise, and PSI-BLAST). The GPkernel also has significantly higher ROC-50 scores than Mismatch and PSI-BLAST (*p *= 0.03 and *p *< 10^-10^), but the GPkernel and SVM-Pairwise's ROC-50 scores are not significantly different (*p *= 0.7). The LA-kernel is, however, the best method in terms of both performance measures (*p *= 0.02 and *p *= 3 * 10^-5 ^for ROC and ROC-50).

**Figure 6:**
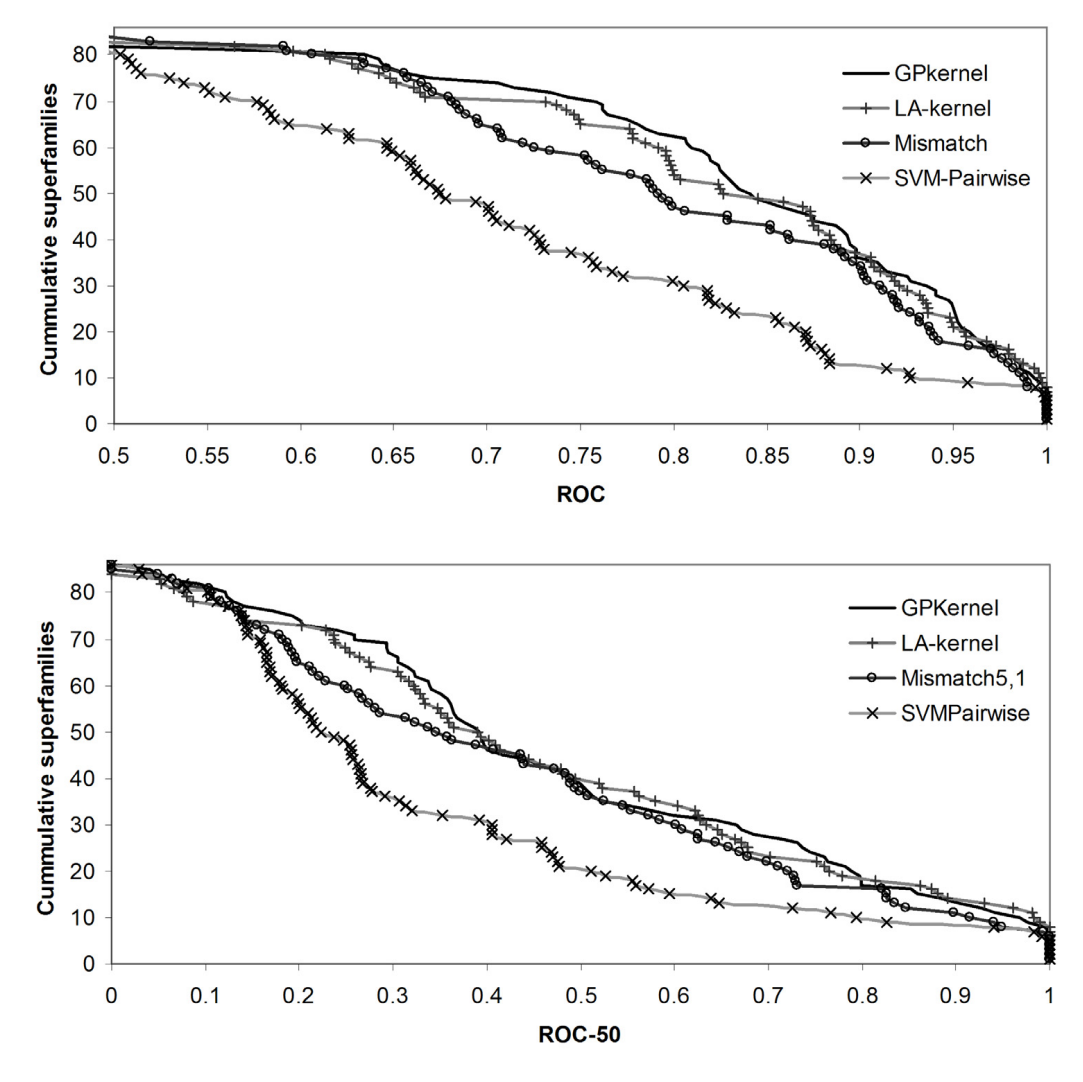
**Large differences in performance on fold detection**. The figure plots the cumulative number of superfamilies with a ROC (top) and ROC-50 (bottom) score greater than a given value for the GPkernel, LA-kernel, Mismatch and SVM-Pairwise methods. PSI-BLAST is omitted in this context due to the method's very poor results on fold detection.

Figure 6 shows how the methods compare on the fold benchmark. As would be expected, there is a bigger difference between the methods when the level of sequence similarity is very low. Especially, the BLAST-based methods have difficulties producing effective alignments between related sequences at the fold level. SVM-Pairwise has a much lower performance on the fold than on the superfamily benchmark, and the scores for PSI-BLAST on fold detection are not reported due to the very poor results achieved. The mismatch kernel, using more general string patterns than the eMOTIF kernel has a stable performance on both benchmarks and is significantly better than SVM-Pairwise on the fold benchmark (*p *= 6 * 10^-8 ^and *p *= 1 * 10^-5 ^for ROC and ROC-50). The GPkernel has the best performance on the fold benchmark. Although the difference to the LA-kernel is not significant when accounting for the multiple testing (*p *= 0.1 and *p *= 0.2 for ROC and ROC-50), the GPkernel is significantly better than the third best performing Mismatch kernel (*p *= 2 * 10^-4 ^and *p *= 7 * 10^-3 ^for ROC and ROC-50). Table 2 gives the average ROC and ROC-50 scores for all of the methods.

**Table 2:** Average ROC/ROC-50 scores on SCOP 1.67 benchmarks

Average ROC and ROC-50 scores				
	Superfamily level		Fold level	
	ROC	ROC-50	ROC	ROC-50

GPkernel	0.902	0.591	0.844	0.514
GPextended	0.869	0.542	0.753	0.371
GPboost	0.797	0.375	0.688	0.298
SVM-Pairwise	0.849	0.555	0.724	0.359
Mismatch	0.878	0.543	0.814	0.467
eMOTIF	0.857	0.551	0.698	0.308
LA-kernel	0.919	0.686	0.834	0.504
PSI-BLAST	0.575	0.175	0.501	0.010

The table shows the average ROC and ROC-50 scores obtained by the different methods on the superfamily benchmark and the fold benchmark.

**Figure 7:**
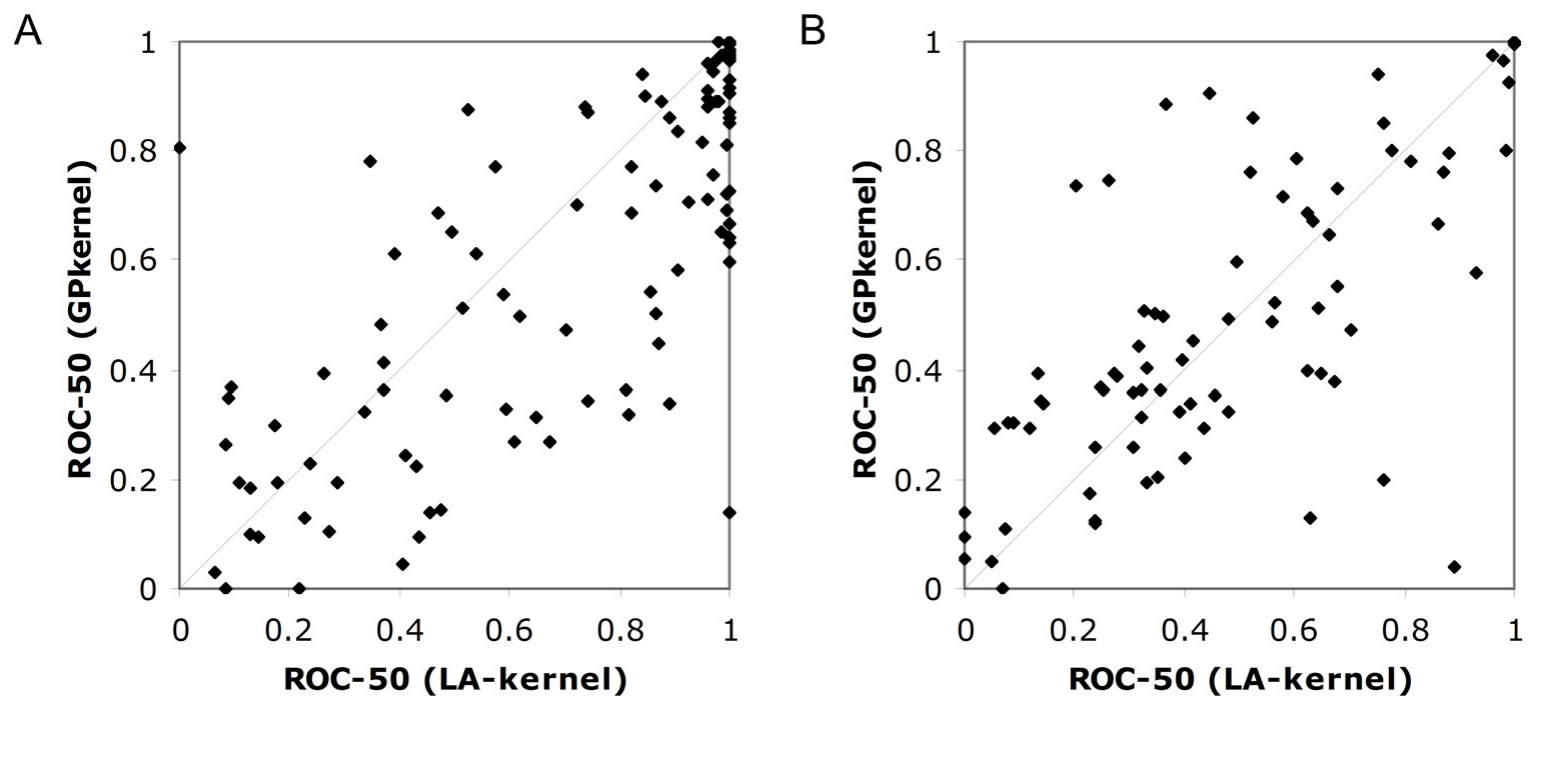
**The GPkernel and LA-kernel predictions are complementary**. The graphs show the LA-kernel's ROC-50 scores plotted against those of the GPkernel on the superfamily (A) and fold (B) benchmarks. Identical predictions from the two methods would fall on the diagonal line.

Although the above results confirm the GPkernel's high performance on the fold benchmark, the results do not prove that the GPkernel is a useful tool for remote homology detection and fold prediction. The GPkernel and LA-kernel have similar performance, and the GPkernel may, for instance, simply make similar predictions as the LA-kernel. If this was the case, one could argue that the GPkernel is a redundant method, as one could simply use the LA-kernel to get the same predictions as the GPkernel. To investigate this, we plotted the GPkernel's ROC-50 score against the LA-kernel's ROC-50 score on all the test sets for the superfamily and fold benchmarks (Figure 7). These graphs show that the two methods are not redundant, but complementary. Although this result may not be surprising – the two methods rely on distinct features – it suggests that the two methods can be combined to create an improved method for remote homology and fold detection [40].

Our SCOP 1.67 benchmark set uses a less stringent sequence similarity filter than the SCOP 1.53 benchmark. To ensure that this higher sequence similarity had not influenced the results for the GPkernel and LA-kernel, we repeated the GPkernel and LA-kernel fold experiments on a modified version of the SCOP 1.67 set. Using the same *E*-value threshold of 10^-25 ^as for the SCOP 1.53 benchmark gave 3511 sequences and 86 superfamilies to use as test sets. The average ROC scores for the GPkernel and LA-kernel were 0.863 and 0.846; the average ROC-50 scores were 0.520 and 0.513. The differences in ROC scores are significant (*p *= 0.009 and *p *= 0.4; ROC and ROC-50).

### Motif based classifiers for fold detection perform better with many motifs of low specificity

One of the SCOP superfamilies (b.68.1) that participate as a test set in the fold detection benchmark is classified well with the GPkernel method (ROC-50 score of 0.903) but achieves a lower score with the eMOTIF method (0.128). Even though there seems to be a mild correlation (0.16) between the number of eMOTIF matches for a fold and the ROC-50 score achieved, the training and test sets for this superfamily do not have significantly fewer eMOTIF matches than other superfamilies. More important is the number of eMOTIFs shared between sequences. This number varies a lot between different pairs of sequences, but if we calculate the average number of eMOTIFs shared between sequences in the b.68 fold, we find that the sequences on average share 0.73 eMOTIFs. This is less than the average for all folds (2.41) which again is much less than the average shared between sequences of a superfamily (11.92). This shows that because sequences at the fold level have a very low sequence similarity, and because of the specificity of most of the eMOTIFs, the number of eMOTIFs shared between sequences in a fold will also be low. This will in turn influence the performance of the eMOTIF kernel.

Table 3 shows examples of GP motifs trained on the training set for the b.68.1 fold classifier. The GP motifs varies and do in general not share any huge similarities with the eMOTIFs that match the sequences of the fold. The table also shows the relative percentage of matches in the positive and negative training and test sets for the fold. The GP motifs do match a higher percentage of the positive sequences than the negatives, but the considerable number of negative sequences that are matched shows the difficulty of finding simple discrete sequence motifs that cover many sequences of a fold while also being as specific as possible. The best GP motifs tend to be either very short sequences or very long complicated expressions with multiple alternative amino acids at each position in the motif.

**Table 3:** Examples of GP motifs

Examples of GP motifs				
Motif	PTr	NTr	PTe	Nte

{*MEEIEII *:*p *>= 3}	67	41	67	55
{*IQIIIEE *: *p *>= 3}	83	38	92	50
{(*I*|*I*)*E*(*E*|(*I*|*E*)) : *p *>= 4}	58	37	83	51
{TQ(*D*|*H*)(*K*|*C*)(*D*|*H*)((((*D*|*H*)|*A*)|*A*)|*A*)*TQ*((*H*|*A*)|*A*)*TQ*((*D*|*H*)|*A*)(*I*|*A*) :*p *>= 7}	33	20	33	23
{*M*(*L*|*L*)*CARACAARAA*(*L*|*L*)*RACAA *: *p *>= 6}	8	28	50	44
{*AALAALA*(*A*|*M*)*AA.ILAL*(*A*|*M*)*AA*(*C*|*M*)*AV.IL*(.|*T*)*A.ILAAALA*(.|(*A*|*M*))	50	28	25	45
*V.ILVAA.ILL*(.|*T*).*IA*(*A*|*M*)*AALA*(*A*|*M*)*V.ILV*(*R*|*M*) : *p *>= 20}				
{(*L*|(*M*|*A*))(*L*|(*L*|(*M*|*A*)))(*L*|(*M*|*A*))(*L*|((*L*|*A*)|*A*))*M *: *p *>= 5}	83	37	67	54

The table shows examples of the motifs evolved by the genetic programming process targeting the SCOP b.68 fold. In addition to amino acid characters, the motifs are also made from the disjunction operator (|), the wildcard operator (.), and the Hamming distance operator {: *p *>= *x*} that specifies the minimum number of characters that must match in the pattern. For each motif, the table shows the relative percentage of sequences matched in the training and test sets. The positive training set has 12 sequences, the negative 3590 sequences. The positive test set also has 12 sequences; the negative set has 226 sequences.

When looking at all motifs made, we find that each motif on average matches nearly a fourth of all sequences. All sequences will therefore share many GP motifs. If we compare the related sequences of a superfamily or fold with randomly chosen sequences, we find that related sequences share more motifs than randomly chosen sequences do. On average, sequences in a superfamily have a higher correlation in their motif matches than other sequences on the positive motifs (correlation coefficient 0.25 versus 0.16) and they also correlate more on the other motifs (0.33 versus 0.21). This means that related sequences share more of all motifs than unrelated sequences, explaining the GPkernel's performance.

Another kernel that also has a good performance on fold detection is the mismatch kernel. This kernel is based on a much larger feature set of even more unspecific patterns than the GPkernel is. For the mismatch kernel, the generality of the patterns ensures that the whole solution space of sequences is covered and that most sequences share at least a few patterns. The GPkernel achieves good coverage by training a certain amount of motifs for each superfamily or fold. The GP motifs, while not being too specific, are still more tuned to discriminate between sequences of different folds than the completely general mismatch *k*-mer patterns. This suggests that to capture the small sequence similarity that exists at the fold level, motif-based classifiers benefit from motifs that are general enough to match a significant number of the weakly similar sequences of a fold. In summary, it seems that good motif-based classifiers on the fold recognition problem need to strike a balance between specificity (eMOTIF) and generality (mismatch). The GPkernel is one step in that direction.

## Conclusion

We have introduced a motif kernel with discrete sequence motifs trained with genetic programming. Motifs are evolved using a subset of regular expressions to describe sequences in a superfamily or fold, and discriminate between these and sequences in other superfamilies (folds). The method gives very good results on two SCOP benchmarks when compared to other relevant methods.

In addition, we have established two new and updated benchmark sets. These sets, which are nearly twice as large as previously used benchmarks, should prove useful for future studies on remote homology detection.

## Methods

### Genetic programming

Genetic programming [27] is a form of automatic programming that aims to find an optimal solution to a problem by using a population of candidate solutions and techniques inspired by biological evolution. In genetic programming, the solutions are usually variable sized syntax trees whose structure is defined by the solution language. An example of such a language is regular expressions where the set of terminals are the 20 amino acid characters.

Our algorithm, which is based on the GP-component of the GPboost algorithm [34], uses a standard tree-based representation of individuals. It uses subtree swapping crossover, tree generating mutation and reproduction as genetic operators and uses tournament selection to select individuals for the next generation.

### Motif kernels

A motif kernel gives a sequence similarity measure based on the motif content of a pair of sequences [19]. A sequence *x *can in this context be represented in a vector space indexed by a set of motifs *M *as Θ(*x*) = (*θ*_*m*_(*x*))_*m*∈*M*_. In the eMOTIF kernel, *θ*_*m*_(*x*) is the number of occurrences of the motif *m *in *x*. The motif kernel is then defined as a linear kernel over the motif contents: *K*(*x*, *x'*) = Θ(*x*) · Θ(*x'*). In most cases a motif appears only once in a sequence so this kernel essentially counts the number of motifs that are common to both sequences. This is always the case for the GPkernel, as *θ*_*m*_(*x*) here is 1 if the motif occurs in *x *and 0 otherwise.

### Results benchmarking

To benchmark our method we simulate the process of remote homology detection and fold detection by using the SCOP database [26] as a basis for two benchmarks. The SCOP database aims to classify all proteins of known structure in a hierarchy based on structural and evolutionary relatedness. At the lowest level of the hierarchy, proteins clustered in a SCOP family have clear evolutionary relationship, meaning that pairwise residue identities between proteins are 30% and greater. Proteins in SCOP superfamilies show low degrees of sequence identities, but structural and functional features in the proteins give them a probable common evolutionary origin, meaning that proteins clustered in superfamilies are likely to be homologues. Proteins have the same common fold if they have the same major secondary structures in the same arrangement and with the same topological connections. This does not necessarily mean they have the same evolutionary origin.

**Figure 8:**
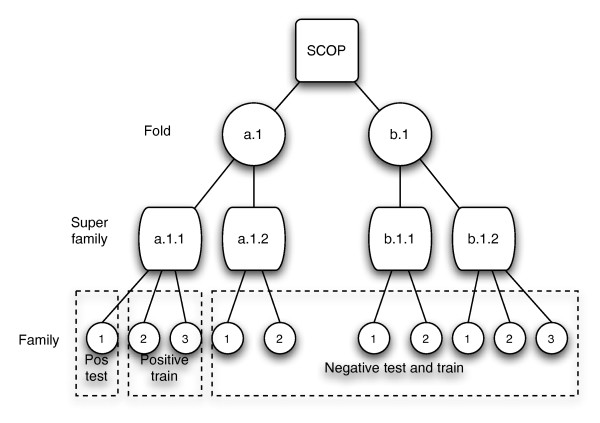
**SCOP superfamily benchmark**. The figure shows how the SCOP database is divided into training and test sets. For each classifier tested on the superfamily benchmark, the sequences of the SCOP database are divided into positive and negative training and test sets. One SCOP family is used as a positive test set. The negative test set is made from one random family from each of the other superfamilies. The positive training set is made from the superfamily of the classifier, excluding the positive test set family. The negative training set is made from the other superfamilies, excluding the negative test sets.

The first benchmark is the now classic benchmark where the goal is to classify a new protein sequence to the correct SCOP superfamily. Here, one family in the superfamily is kept as a positive test set. The other families in the same superfamily constitute the positive training set. The negative test set consists of one random family from each of the other superfamilies and the negative training set has the rest of the families in these superfamilies. Figure 8 illustrates the setup for this benchmark.

For the other benchmark, we follow that of Rangwala and Karypis [18]. We move up one level in the SCOP hierarchy; the objective is to classify an unknown sequence to the correct fold. One superfamily is used as positive test set, while the others in the same fold constitute the positive training set. The negative test set consists of one random superfamily from each of the other folds, and the negative training set consists of the remaining sequences. This benchmark is considerably harder than the superfamily benchmark is, as most of the sequences within a fold have a very low degree of similarity.

We use sequences from SCOP version 1.67, filtered with Astral [41] to remove sequences that share more than 95% similarity. The data are further filtered according to the principle that each classifier should have at least 10 sequences for testing and training, that is, every classifier should have at least 10 sequences in its positive training and test set. For the superfamily benchmark, this leaves us with 4019 sequences in 392 SCOP families. 102 of these families match the conditions above. The fold benchmark has 3840 sequences from 374 superfamilies and classifiers are made for 86 of these. Of the 3840 sequences in the fold benchmark, 2076 do not participate in the superfamily benchmark. Note that the 102 families and 86 superfamilies tested in our superfamily and fold benchmarks are almost twice the number of families and superfamilies used in previous benchmark studies.

Our benchmark sets are available as online supplementary material (Additional files 1, 2, 3).

### Performance measures

Because the test sets have many more negative than positive instances, simply measuring error-rates will not give a good evaluation of performance. Instead we evaluate our results by computing the ROC and ROC-50 scores [42]. A ROC curve is a plot of a classifier's sensitivity as a function of its specificity for different classification thresholds. The ROC score is the area under the ROC curve. The ROC-50 curve is the same as a ROC curve, except the curve only shows the classifier's sensitivity for the first 50 negatives.

### Statistical tests

To determine whether two methods have statistically different ROC or ROC-50 scores on a particular benchmark, we use signed rank tests. All *p*-values reported are double-sided *p*-values that have been Bonferroni-corrected for multiple comparisons.

### Other methods

We computed the eMOTIF kernel based on the eMOTIFs generated from version 1.0 of the eBLOCKS database. This database contains 522,321 motifs and is the same that was used in the original article [19]. The mismatch kernel is computed by extracting all subsequences of length 5 from the dataset and using the Interagon PMC to search for these subsequences in the data sets, allowing for one mismatch. We use Clustal W [43] version 1.83 to create a multiple alignment of the positive training set and give this as input to PSI-BLAST version 2.2.13. PSI-BLAST is then run with standard parameter values for 1 iteration against the test set. The e-value of the resulting alignments are used to rank the test set. SVM-pairwise is calculated by using the negative logarithm of pairwise BLAST e-values to generate a radial basis kernel with the same parameters as in the original article of Liao and Noble [12]. We use the authors' default implementation [44] to compute the LA-kernel.

The Gist package version 2.2 [45] is used to train and test the kernels.

## Authors' contributions

TH did the experiments and drafted the manuscript. AJHH did the initial work on GPboost for remote homology detection. PS conceived the GPkernel and the study and helped prepare the final version of the manuscript. All authors read and approved the final manuscript.

## Supplementary Material

Additional file 1 The SCOP 1.67 superfamily benchmark filtered by 95% sequence similarity. The file is a tab-delimited table. The first column gives SCOP sequence IDs; the first row identifies the positive test set for a particular benchmark instance; values (1, 2, 3, or 4) identify whether the sequence is in the positive test, negative test, positive training, or negative training sets. Click here for file

Additional file 2 The SCOP 1.67 fold benchmark filtered by 95% sequence similarity. The file has the same format as Additional file 1. Click here for file

Additional file 3 The SCOP 1.67 fold benchmark filtered by an E-value threshold of 10^-25 ^The file has the same format as Additional file 1. Click here for file
